# The Impact of Short-Term Outdoor Air Pollution on Clinical Status and Prognosis of Hospitalized Patients with Coronary Artery Disease Treated with Percutaneous Coronary Intervention

**DOI:** 10.3390/jcm11030484

**Published:** 2022-01-18

**Authors:** Piotr Desperak, Aneta Desperak, Bożena Szyguła-Jurkiewicz, Piotr Rozentryt, Andrzej Lekston, Mariusz Gąsior

**Affiliations:** 1 Third Department of Cardiology, Faculty of Medical Sciences in Zabrze, Medical University of Silesia, 40-752 Katowice, Poland; acis777@gmail.com (A.D.); centrala4@wp.pl (B.S.-J.); jerzyrozentryt@poczta.onet.pl (P.R.); alekstonhemo@sccs.pl (A.L.); mgasior@sum.edu.pl (M.G.); 2 Department of Toxicology and Health Protection, Faculty of Health Sciences in Bytom, Medical University of Silesia in Katowice, 41-902 Bytom, Poland

**Keywords:** acute coronary syndromes, air pollutions, chronic coronary syndromes, percutaneous coronary intervention

## Abstract

Background: The aim of this study was to determine the influence of acute exposure to air pollutants on patients’ profile, short- and mid-term outcomes of hospitalized patients with coronary artery disease (CAD) treated with coronary angioplasty. Methods: Out of 19,582 patients of the TERCET Registry, 7521 patients living in the Upper Silesia and Zaglebie Metropolis were included. The study population was divided into two groups according to the diagnosis of chronic (CCS) or acute coronary syndromes (ACS). Data on 24-h average concentrations of particulate matter with aerodynamic diameter <10 μm (PM10), sulfur dioxide (SO_2_), nitrogen monoxide (NO), nitrogen dioxide (NO_2_), and ozone (O_3_) were obtained from eight environmental monitoring stations. Results: No significant association between pollutants’ concentration with baseline characteristic and in-hospital outcomes was observed. In the ACS group at 30 days, exceeding the 3rd quartile of PM10 was associated with almost 2-fold increased risk of adverse events and more than 3-fold increased risk of death. Exceeding the 3rd quartile of SO_2_ was connected with more than 8-fold increased risk of death at 30 days. In the CCS group, exceeding the 3rd quartile of SO_2_ was linked to almost 2,5-fold increased risk of 12-month death. Conclusions: The acute increase in air pollutants’ concentrations affect short- and mid-term prognosis in patients with CAD.

## 1. Introduction

Air pollution is a major health problem in developing as well as in developed countries. Despite the preventive actions, the World Health Organization’s target concentrations of the pollutants are not always achieved [1]. It is a challenge, especially in highly urbanized and industrialized territories. The influence of air pollution on the manifestation and exacerbation of respiratory diseases and mortality is well-documented [2,3,4,5,6]. Data from RACE PARIS registry suggest that even in such a healthy group as long-distance runners, air pollution may trigger major cardiac events during the race [2]. A large study performed in the USA have shown that particulate matter and SO_2_ pollution were associated with all-cause, lung cancer, and cardiopulmonary mortality [3]. Moreover, an increased chronic and short-term concentration of air pollutants is associated with more frequent manifestations of cardiovascular diseases. In some large-population registries and meta-analyses, the relationship between worse air quality and coronary artery disease (CAD) has been shown [7,8,9,10,11,12,13,14,15,16,17].

However, contemporary studies have focused mainly on the incidence of acute myocardial infarction (MI) [8,9,10,12,13]. Except for a single retrospective study [13], the relationship between air pollution and the patients’ characteristics, the severity of the clinical status, and follow-up outcomes were not analyzed. English studies performed on an acute coronary syndromes’ registry have shown the relationship of long-term exposure on particulate matter, and oxidant gases with mortality and re-admissions after acute coronary syndromes. However, in mentioned studies there are no data on short-term exposition on air pollutants [14,15]. Moreover, there is a lack of studies evaluating patients with chronic coronary syndromes (CCS) in the aspect of air pollution [10]. We hypothesize short-term increases in air pollution concentrations may affect not only the incidence, but also the more severe clinical course of CAD.

The purpose of the present study was to determine the influence of acute exposure to air pollutants on patients’ profiles and short- and mid-term outcomes of hospitalized patients with CAD treated with a percutaneous coronary intervention (PCI).

## 2. Materials and Methods

### 2.1. The TERCET Registry

The design of the Hyperlipidemia Therapy in Tertiary Cardiological Center (TERCET) registry (ClinicalTrials.gov ID: NCT0306543) was described previously [18]. Briefly, the TERCET registry is a prospective, observational study recruiting consecutive patients with CCS and acute coronary syndromes (ACS) hospitalized in a tertiary cardiology center with an on-site cardiac surgery facility. Diagnostic and therapeutic strategies, including pharmacologic and interventional treatment, were used in accordance with the European Society of Cardiology (ESC) guidelines [19,20,21].

The data on long-term follow-up, including the exact date of death and cardiovascular events, were obtained from the official registry of the National Health Fund. Follow-up was available for all patients. The TERCET registry was approved by the institutional review board and was conducted in accordance with the ethical standards laid down in the 1964 Declaration of Helsinki and its latest revision.

### 2.2. Study Population

The present study includes data of inhabitants of the Upper Silesia and Zaglebie Metropolis (USZM). The place of residence was determined based on contact details from the medical history. USZM is a conurbation located in the Silesian Voivodship around the City of Katowice with an area of 2554 km^2^ and a population of over 2.2 million people [22].

Of 19,582 patients of the TERCET Registry hospitalized between 2006 and 2016, 13,676 patients living in USZM were included. Further analysis considered 7521 patients who underwent PCI. The study population was divided into two groups according to the diagnosis of CCS or ACS.

### 2.3. Air Pollutants

For the air pollution exposure estimate, the 24-h average concentrations of particulate matter with aerodynamic diameter < 10 μm (PM10), sulfur dioxide (SO_2_), nitrogen monoxide (NO), nitrogen dioxide (NO_2_), and a daily maximum of 8-h mean of ozone (O_3_) on the day of hospitalization were considered. Data on daily average concentrations of the pollutants were obtained from the Silesian Inspectorate of Environmental Protection public archive for eight environmental monitoring stations. The values were averaged between the stations for each day. Particulate matter concentrations were measured using the gravimetric method, whereas gaseous pollutants were measured with the use of the automated method.

### 2.4. Definitions

The definitions of CCS and ACS were established based on clinical presentation, coronary angiography, and additional test results, in accordance with the current ESC guidelines for the given period [19,20,21]. ACS included ST-segment elevation MI, non-ST-segment elevation MI, and unstable angina. In-hospital death was defined as the index hospitalization finished according to patient′s death. In-hospital MI included periprocedural or in-hospital MI according to the current universal definition of myocardial infarction for the index hospitalization period. Cardiac arrest was defined as the sudden cessation of cardiac activity so that the victim becomes unresponsive, with no normal breathing and no signs of circulation. The major adverse cardiovascular or cerebrovascular events (MACCE) was defined as the occurrence (at least one of) of death from any cause, non-fatal MI, ACS-driven PCI, or stroke. Non-fatal MI included periprocedural or in-hospital MI according to the current universal definition of myocardial infarction for the given period [23]. ACS-driven PCI was defined as acute myocardial ischemia (myocardial infarction or unstable angina) requiring urgent PCI or coronary artery bypass graft performed as an urgent procedure because of acute ischemic symptoms [24]. The heating season (a term in Polish legislation regulated by the Minister of Economy) is defined as a period in which the weather conditions force the supply of heat to buildings continuously. In the most of highly urbanized regions, heating season is connected with intensified heating of low-rise houses, which is a trigger for increase of air pollutants concentrations. For the purposes of the present analysis, the heating season was defined as the annual period from 1 October to 30 March.

### 2.5. Statistical Analysis

The characteristics of the groups were expressed as median with first and third quartiles for continuous variables and as percentages for categorical data. The normality of quantitative data distributions was checked with the Shapiro–Wilk test. Continuous variables following the normal distribution were compared using the Student’s t-test, while other than normal were compared using the Mann–Whitney U test. Qualitative data were analyzed using the Pearson Chi-squared test. The association of air pollutants and in-hospital outcomes was calculated with logistic regression, the results were expressed as odds ratios (ORs) and 95% confidence intervals (95% CIs). Factors influencing the 30-day and 12-month outcomes were analyzed using a Cox proportional regression model. As a first step, the bivariate analysis of air pollutants and candidate covariates was performed. Then, in the multivariate analysis, we used the enter regression method. The criterion to enter the analysis was *p* < 0.30 at univariate analysis for both air pollutants and candidate covariates. The heating season and year of the admission were entered regardless of the *p*-value. The results are presented as hazard ratios (HRs) and 95% confidence intervals (CIs). The statistical significance level was set to *p* < 0.05. All calculations were made in TIBCO Software Inc. (2017), Statistica (data analysis software system), version 13, http://statistica.io.

## 3. Results

The descriptive statistics of the air pollutants’ concentrations are shown in Table 1. In the CCS group there were 3347 patients and in the ACS group there were 174 patients. Characteristics of the total, ACS, and CCS groups in two following subgroups are shown in Table 1: (1) admitted when at least one of the air pollutants exceeded the third quartile and (2) admitted when all of the air pollutants were below the third quartile. The descriptive statistics of the air pollutants’ concentrations are presented in Table 2. The concentrations of PM10 and SO_2_ were significantly higher and the O_3_ concentration was lower in the ACS group. No significant association between pollutants’ concentrations and in-hospital outcomes in both the ACS and CCS groups was observed (Supplementary Table S1).

In the multivariable analysis of the ACS group, exceeding the 3rd quartile of PM10 was associated with almost 2-fold increased risk of MACCE at 30 days (HR: 1.97; 95% CI: 1.03–3.77; *p* = 0.042) and more than 3-fold increased risk of death from any cause at 30 days (HR: 3.23; 95% CI: 1.08–9.67; *p* = 0.036). Moreover, in this group exceeding the 3rd quartile of SO_2_ was connected with a more than 8-fold increased risk of death from any cause at 30 days (HR: 8.71; 95% CI: 2.25–33.67; *p* = 0.0017). In the CCS group, only exceeding the 3rd quartile of SO_2_ was linked to over 2-fold increased risk of 12-month death from any cause (HR: 2.29; 95% CI: 1.03–5.11; *p* = 0.043). In the CCS group exceeding the 3rd quartile of O_3_ was linked with lower risk of myocardial infarction at 12 months (HR: 0.42; 95% CI: 0.19–0.92; *p* = 0.031). The association of air pollutants and percutaneous coronary intervention, or stroke was not found. The results are presented in Table 3.

## 4. Discussion

To our knowledge, this is the first observational study of such a specific population of patients with coronary syndromes treated with PCI, living in a highly urbanized and polluted area. The major advantage of this analysis is taking into account in-hospital, short-term, and mid-term outcomes of acute exposure to air pollutants at the day of admission. It is worth emphasizing that a significant increase in ambient air pollutants’ concentrations on the day of admission could have contributed to worse clinical status and course of the disease. We did not observe a significant association of air pollution with in-hospital outcomes; however, in our study, the impact of PM10 and SO_2_ on the 30-day follow-up was demonstrated.

### 4.1. Incidence of Acute Coronary Syndromes

In our analysis, higher concentrations of PM10 and SO_2_ at the day of hospitalization with acute coronary syndromes were observed than in patients hospitalized with chronic coronary syndromes. Numerous studies confirm the dependence between worse air conditions and a higher occurrence of MI [8,9,10,12,13,14]. The metanalysis of 34 studies by Mustafic et al. showed the significant association of elevated concentrations of nitrogen oxides, CO, SO_2_, and particulate matter with a higher occurrence of MI [8]. Buszman et al. indicated that the most important pollutants triggering acute myocardial infarction in Southern Poland are particulate matters and gaseous pollutants including NO_2_ and SO_2_ [12]. In a large analysis of emergency department visits in Canadian cities, Stieb et al. confirmed a strong correlation between NO_2_ and coronary artery disease exacerbation including unstable angina and MI; however, this population was in contrast to our study hospitalized in emergency department over a decade earlier (1990–2000), and data were analyzed according to Poisson Regression model [4]. This effect was more noticeable in the elderly with comorbidities such as arterial hypertension [25]. In our analysis, we have not confirmed the association between nitric oxides and acute coronary syndromes; however, it may be the result of differences between study populations and study designs.

### 4.2. Pathophysiology

Air pollution is a well-recognized risk factor for atherosclerosis. Many studies document the relationship between elevated concentrations of particulate ambient air pollution and higher intima-media thickness, aortic, and coronary arteries calcifications [26,27,28,29]. The prothrombotic effect of air pollutants was shown in connection with higher plasma viscosity, shorter prothrombin time, and higher platelets count [30,31,32,33]. Other possible mechanisms include oxidative stress, systemic inflammation, and influence on the autonomic system [31,33,34]. Moreover, some studies suggest that elevated levels of ambient air pollutants may promote ST-segment changes in patients with coronary artery disease [33,35,36,37]. These possible mechanisms could explain the role of air pollution as a trigger of acute coronary syndromes.

### 4.3. Adverse Cardiovascular Events

Air pollutants are risk factors of mortality, which has been established in multiple studies [3,5,7,38]. In their analysis, Xie et al. observed the association of higher concentrations of PM10 with sudden cardiac death occurrence [10]. In the study performed on the population of Silesian cities, Dziubanek et al. found that reduction of annual average PM10 concentration by 10 μg/m^3^ may extend the length of life by 0.1 year [39]. Pope et al. estimated that a 10 μg/m^3^ PM2.5 decrease may result in a 0.61 ± 0.2-year extension of life expectancy [38]. PM2.5 was not used in our study; however, it is noteworthy that 50–70% of PM10 mass is consisted of PM2.5. [40] In the ACS subgroup of the study population, we observed an almost two times higher risk of 30-day composite outcome including a three times higher risk of 30-day death when the concentrations of PM10 exceeded the 3rd quartile, and over eight times higher when the concentrations of the SO_2_ exceeded the 3rd quartile. These results appear to be high, especially in the case of SO_2_. However, there are no comparable studies available to verify these findings. Despite using the most important factors to adjust the impact of the pollutants to the outcomes, there still remain many other confounders that might not be available to the analysis. Therefore, these results should be interpreted with caution.

### 4.4. Ozone Phenomenon

Contrary to previous analyses [8,41], we noticed a connection between the ACS hospitalizations and lower O_3_ concentration than in the case of CCS hospitalizations. In the multivariable analysis, exceeding 3rd quartile of O_3_ concentration on the day of admission with CCS was a protective factor for myocardial infarction at 12 months. In some studies, the protective effect of O_3_ on cardiovascular events was also observed [42,43]. This phenomenon could be explained by a negative correlation of O_3_ with a pollutant that was not considered [44]. O_3_ is also a highly reactive molecule, and it can be both substrate and product of photochemical reactions between other pollutants, which could lead to differences between monitoring station′s records and real exposure.

### 4.5. Study Limitations

The results of the present study must be interpreted with caution, due to few implications derived from its retrospective nature. To reflect average air pollutants concentrations and considering that Polish cities are some of the most polluted in Europe, we decided to split pollutants’ concentrations into four quartiles and to compare 25% of days with the highest AP (above Q3) concentrations with remaining 75% (below Q3). This type of division does not allow for the assessment of continuous pollutant values, but it allows us to signal that, despite meeting the WHO standards, high pollutant concentrations are still associated with adverse cardiovascular events. Our study indicates the association between some of the air pollutants′ levels and adverse events after PCI. However, the results of the analysis may not reflect individual exposure. We did not take into account data that include patients living in rural areas. On the other hand, we analyzed a specific population concentrated in an urbanized region of a relatively small area and homogeneous landform features, which may be especially exposed to potential adverse effects of the pollutants. In some cases, high sample size could be related with significant *p*-value without a clinical difference (i.e., value of hemoglobin in both groups). The results of our study should be confirmed by conducting more analyses. There is a need to assess the short-term impact of air pollutants including moving averages and lags. Therefore, we consider the presented results as preliminary.

## 5. Conclusions

Summarizing the presented results of 7521 consecutive patients with CCS or ACS, we concluded the following: (1) the PM10 and SO_2_ concentrations are higher and O_3_ concentrations are lower during ACS hospitalization in comparison to CCS; (2) no connection between in-hospital outcomes and elevated levels of air pollutants was observed; (3) however, after adjustment, the relationship between higher concentrations of particulate matters and SO_2_ with short-term outcomes in the ACS group and mid-term mortality in CCS group was shown. Due to the limitations of the study, the conclusions should be considered with caution. However, the results indicate that an acute increase of concentrations of air pollutants not only quantitatively affects the number of ACS hospitalizations, but also in the case of their occurrence is associated with a worse short- and mid-term prognosis. Moreover, there is still a need to perform more detailed studies to assess the impact of air quality on the health of susceptible subgroups of patients such as those with coronary artery disease treated with percutaneous coronary intervention.

## Figures and Tables

**Table 1:** Clinical characteristics of total sample and study groups.

Factor	Total *n* = 7521	CCS *n* = 3347			ACS *n* = 4174		
		AP ≤ Q3 *n* = 1345	AP > Q3 *n* = 2002	*p*	AP ≤ Q3 *n* = 1645	AP > Q3 *n* = 2529	*p*
Age, years; median (Q1–Q3)	65 (58–73)	65 (59–73)	66 (59–73)	0.69	64 (57–73)	65 (57–73)	0.70
BMI, kg/m^2^; median (Q1–Q3)	28.1 (25.4–31.2)	28.1 (25.5–31.1)	28.1 (25.4–31.2)	0.81	27.8 (25.3–31.2)	28.1 (25.4–31.2)	0.29
Female, %	31.1	32.1	29.2	0.07	32.3	31.9	0,67
Prior MI, %	37.4	45.3	47.4	0.24	30.8	29.7	0.44
Arterial hypertension, %	75.0	82.9	81.1	0.19	68.4	70.5	0.15
COPD, %	6.1	6.2	6.5	0.66	6.3	5.3	0.31
Atrial fibrillation, %	12.6	13.0	15.0	0.11	12.6	10.4	0.034
Diabetes mellitus, %	34.8	38.6	38.6	0.98	31.6	31.9	0.83
Dyslipidemia, %	66.4	77.0	77.1	0.94	56.4	59.0	0.10
PAD, %	11.2	14.0	17.7	0.005	6.8	7.6	0.32
Obesity, %	28.8	29.1	29.9	0.64	27.5	28.0	0.80
History of cigarette smoking, %	46.7	49.6	48.9	0.68	43.8	45.2	0.39
Stenocardial pain, %	78.4	67.2	66.3	0.60	92.0	91.9	0.91
SBP *, mmHg; median (Q1–Q3)	135 (120–150)	130 (120–140)	130 (120–140)	0.032	140 (130–163)	140 (130–162)	0.85
DBP *, mmHg; median (Q1–Q3)	80 (70–90)	80 (70–85)	80 (70–80)	0.008	80 (72–92)	80 (74–95)	0.47
HR *, mmHg; median (Q1–Q3)	70 (65–80)	70 (64–77)	70 (64–80)	0.41	75 (68–85)	75 (67–85)	0.25
LVEF *, %; median (Q1–Q3)	47 (40–52)	50 (43–55)	50 (40–55)	0.28	45 (38–50)	45 (38–50)	0.46
WBC *, thousand/mm^3^; median (Q1–Q3)	8.2 (6.6–10.3)	7.3 (6.1–8.8)	7.2 (6.0–8.6)	0.11	9.1 (7.4–11.9)	9.3 (7.3–11.9)	0.75
HCT *, %; median (Q1–Q3)	41.3 (38.3–43.8)	41.2 (38.6–43.6)	41.0 (38.2–43.4)	0.16	41.3 (38.3–44.0)	41.5 (38.1–44.1)	0.45
Hemoglobin *, mmol/L; median (Q1–Q3)	8.7 (8.1–9.3)	8.8 (8.1–9.3)	8.7 (8.1–9.2)	0.012	8.8 (8.1–9.4)	8.8 (8.0–9.4)	0.99
Serum creatinine *, µmol/L; median (Q1–Q3)	82.0 (69.5–98.8)	80.5 (68.6–97.0)	81.7 (69.5–98.0)	0.16	83.4 (69.9–100.0)	82.0 (69.7–100,0)	0.39
Glucose *, mmol/L; median (Q1–Q3)	6.4 (5.5–8.3)	5.8 (5.2–7.1)	5.9 (5.2–7.3)	0.52	7.0 (5.8–9.0)	6.9 (5.8–9.1)	0.99
Multivessel CAD, %	51.8	51.3	50.9	0.82	53.0	51.9	0.46
Stent implantation, %	88.7	89.9	89.5	0.73	88.8	87.3	0.14
DES, %	60.9	65.3	64.3	0.56	58.3	57.6	0.66
In-hospital outcomes							
Death, %	2.2	1.1	0.6	0.15	3.5	3.2	0.60
MI, %	1.1	1.0	0.4	0.07	1.3	1.5	0.62
Cardiac arrest, %	3.5	1.4	0.8	0.09	5.3	5.6	0.63
Pulmonary oedema, %	2.5	0.1	0.1	0.66	4.9	4.0	0.16
Cardiogenic shock, %	2.5	0.1	0.1	0.65	4.9	4.1	0.26
30-day MACCE, %	5.3	3.3	2.3	0.08	7.1	7.5	0.63
Death, %	2.8	1.0	1.0	0.98	4.1	4.4	0.71
MI, %	2.2	1.8	1.1	0.13	3.0	2.6	0.51
ACS-driven PCI, %	1.1	1.0	0.7	0.50	1.1	1.5	0.31
Stroke, %	0.1	0.1	0.0	0.84	0.1	0.2	0.67
12-month MACCE, %	11.8	7.8	7.5	0.74	15.5	14.9	0.63
Death, %	5.4	2.9	3.3	0.52	7.2	7.2	0.96
MI, %	4.9	3.1	2.9	0.77	6.9	6.2	0.35
ACS-driven PCI, %	4.2	3.2	3.0	0.74	5.4	4.8	0.40
Stroke, %	0,7	0.7	0.6	0.66	0.5	0.9	0.12

* on admission; The subgroups AP > Q3—at least one of the air pollutants exceeds the third quartile. Abbreviations: ACS, acute coronary syndrome; AP, air pollutants; BMI, body mass index; CCS, chronic coronary syndrome; DBP, diastolic blood pressure; DES, drug-eluting stent; COPD, chronic obstructive pulmonary disease; HCT, hematocrit; HR, heart rate; LVEF, left ventricular ejection fraction; MACCE, major cardiac and cerebrovascular events; MI, myocardial infarction; PAD, peripheral artery disease; PCI, percutaneous coronary intervention; SBP, systolic blood pressure; WBC, white blood cells.

**Table 2:** Air pollutants during day of admission to the hospital in study groups.

Factor	WHO Recommended Level 2021 (vs. 2005)	Study Population *n* = 7494	CCS *n* = 3347	ACS *n* = 4174	*p* Value
PM10, µg/m^3^; median (Q1–Q3)	15 (20)	36.2 (25.8–54.4)	35.4 (25.8–53.4)	36.8 (25.8–56.0)	0.039
SO_2_, µg/m^3^; median (Q1–Q3)	40 (20)	11.4 (7.5–19.5)	10.9 (7.4–18.6)	11.8 (7.6–20.2)	0.0001
NO, µg/m^3^; median (Q1–Q3)	*n*/a	7.7 (4.2–16.2)	7.8 (4.4–15.8)	7.7 (4.0–16.4)	0.17
NO_2_, µg/m^3^; median (Q1–Q3)	25 (40)	24.7 (18.5–32.2)	24.6 (19.0–32.0)	24.8 (18.2–32.5)	0.51
O_3_, µg/m^3^; median (Q1–Q3)	100 (100)	60.7 (37.0–84.7)	62.7 (39.3–86.3)	59.0 (35.3–83.7)	0.0001

Abbreviations: NO, nitrogen monoxide; NO_2_, nitrogen dioxide; O_3_, daily maximum of 8-h mean of ozone; PM10, particulate matter with aerodynamic diameter < 10 µm; SO_2_, sulphur dioxide; WHO—World Health Organization.

**Table 3:** The influence of exceeding 3rd quartiles of air pollutants on short- and mid-term outcomes in the chronic coronary syndromes (CCS; **A**) and acute coronary syndromes (ACS; **B**) subgroup. Abbreviations: BMI, body mass index; CAD, coronary artery disease; CCS, chronic coronary syndromes; EF, left ventricular ejection fraction; MACCE, major adverse cardiovascular or cerebrovascular events; MI, myocardial infarction; NO, nitric oxide; NO_2_, nitric dioxide; O_3_, ozone; PM10, particulate matter with aerodynamic diameter < 10 um; SBP, systolic blood pressure; SO_2_, sulfur dioxide; U-PCI, urgent percutaneous coronary intervention; WBC, white blood cells count.

**A**					**CCS**					
	**PM10**		**TsSO_2_**		**NO**		**NO_2_**		**O_3_**	
	**HR (95% CI)**	***p***	**HR (95% CI)**	***p***	**HR (95% CI)**	***p***	**HR (95% CI)**	***p***	**HR (95% CI)**	***p***
30d MACCE	1.15 (0.52–2.56)	0.73	1.54 (0.63–3.80)	0.33	1.48 (0.68–3.23)	0.32	1.05 (0.47–2.33)	0.91	0.46 (0.15–1.39)	0.17
Covariates used in the model: year of the admission, age, atrial fibrillation, chest pain, creatinine, EF, hemoglobin, heating season, multivessel CAD, WBC, prior MI										
30-day mortality	1.31 (0.52–3.31)	0.56	1.26 (0.45–3.53)	0.66	0.85 (0.36–2.04)	0.75	1.14 (0.47–2.78)	0.76	0.83 (0.35–2.00)	0.68
Covariates used in the model: year of the admission, age, BMI, creatinine, EF, heating season, multivessel CAD, prior MI, SBP, WBC										
30-day MI	1.27 (0.63–2.55)	0.5	1.19 (0.54–2.60)	0.67	0.73 (0.35–1.50)	0.39	1.03 (0.51–2.08)	0.94	0.47 (0.18–1.19)	0.11
Covariates used in the model: year of the admission, cardiogenic shock, heating season, prior MI										
30-day stroke	-	-	-	-	-	-	-	-	-	-
not enough events to perform the multivariable analysis										
30-day ACS-driven PCI	1.94 (0.73–5.17)	0.18	2.95 (0.90–9.71)	0.07	1.41 (0.55–3.61)	0.47	1.41 (0.53–3.73)	0.49	0.49 (0.13–1.78)	0.28
Covariates used in the model: year of the admission, age, chest pain, creatinine, hemoglobin, heating season, prior MI										
12-month MACCE	0.88 (0.52–1.49)	0.63	1.25 (0.71–2.20)	0.44	1.06 (0.65–1.72)	0.83	1.22 (0.76–1.98)	0.41	0.76 (0.43–1.34)	0.34
Covariates used in the model: year of the admission, age, BMI, cardiogenic shock, chest pain, creatinine, EF, hemoglobin, heating season, multivessel CAD, prior MI, WBC										
12-month mortality	1.58 (0.78–3.18)	0.2	2.29 (1.03–5.11)	0.043	0.99 (0.51–1.94)	0.98	1.37 (0.71–2.65)	0.34	0.82 (0.38–1.72)	0.59
Covariates used in the model: year of the admission, age, creatinine, BMI, cardiogenic shock, cigarette smoking, EF, heating season, multivessel CAD, prior MI, SBP, WBC										
12-month MI	1.08 (0.56–2.06)	0.81	1.09 (0.54–2.24)	0.8	0.92 (0.50–1.69)	0.79	1.20 (0.65–2.21)	0.56	0.42 (0.19–0.92)	0.03086
Covariates used in the model: year of the admission, BMI, cardiogenic shock, cigarette smoking, heating season, EF, hemoglobin, multivessel CAD, prior MI, SBP										
12-month stroke	0.41 (0.11–1.52)	0.18	1.26 (0.37–4.29)	0.71	0.51 (0.17–1.54)	0.23	0.75 (0.26–2.18)	0.6	1.54 (0.60–3.94)	0.36
Covariates used in the model: year of the admission, age, atrial fibrillation, chest pain, diabetes mellitus, EF, female, hemoglobin, heating season, prior MI, WBC										
12-month ACS-driven PCI	1.07 (0.71–1.64)	0.71	0.90 (0.55–1.45)	0.66	1.23 (0.84–1.78)	0.28	1.02 (0.69–1.53)	0.91	0.93 (0.62–1.41)	0.75
Covariates used in the model: year of the admission, diabetes mellitus, heating season, multivessel CAD, prior MI, SBP.										
**B**					**ACS**					
	**PM10**		**SO_2_**		**NO**		**NO_2_**		**O_3_**	
	**HR (95% CI)**	***p***	**HR (95% CI)**	***p***	**HR (95% CI)**	***p***	**HR (95% CI)**	***p***	**HR (95% CI)**	***p***
30d MACCE	1.97 (1.03–3.77)	0.0419	2.07 (0.95–4.53)	0.07	1.52 (0.82–2.86)	0.18	1.35 (0.70–2.64)	0.36	0.98 (0.48–2.01)	0.97
Covariates used in the model: year of the admission, atrial fibrillation, BMI, cardiogenic shock, chest pain, cigarette smoking, creatinine, EF, hemoglobin, heating season, multivessel CAD, prior MI, SBP, WBC										
30-day mortality	3.23 (1.08–9.67)	0.036	8.71 (2.25–33.67)	0.0017	1.33 (0.48–3.68)	0.58	2.34 (0.82–6.70)	0.11	1.13 (0.35–3.63)	0.84
Covariates used in the model: year of the admission, age, BMI, cardiogenic shock, chest pain, cigarette smoking, creatinine, diabetes mellitus, EF, hemoglobin, heating season, multivessel CAD, prior MI, SBP, WBC										
30-day MI	0.88 (0.57–1.37)	0.58	0.98 (0.64–1.50)	0.92	0.85 (0.54–1.33)	0.48	0.76 (0.48–1.20)	0.24	0.97 (0.62–1.52)	0.9
Covariates used in the model: year of the admission, age, cigarette smoking, diabetes mellitus, EF, multivessel CAD, prior MI										
30-day stroke	-	-	-	-	-	-	-	-	-	-
not enough events to perform the multivariable analysis										
30-day ACS-driven PCI	1.51 (0.67–3.42)	0.31	1.73 (0.67–4.52)	0.25	1.25 (0.57–2.76)	0.58	1.06 (0.45–2.49)	0.89	0.97 (0.36–2.56)	0.95
Covariates used in the model: year of the admission, diabetes mellitus, heating season, multivessel CAD, prior MI, SBP										
12-month MACCE	1.14 (0.79–1.62)	0.48	1.11 (0.74–1.67)	0.62 (	1.06 (0.75–1.50)	0.74	0.87 (0.60–1.27)	0.47	1.24 (0.84–1.84)	0.27
Covariates used in the model: year of the admission, age, atrial fibrillation, BMI, cardiogenic shock, chest pain, cigarette smoking, creatinine, EF, hemoglobin, heating season, prior MI, SBP, WBC										
12-month mortality	1.12 (0.66–1.91	0.67	1.11 (0.60–2.06)	0.72	0.90 (0.54–1.50)	0.69	1.12 (0.66–1.91)	0.67	0.88 (0.52–1.51)	0.65
Covariates used in the model: year of the admission, age, atrial fibrillation, BMI, creatinine, chest pain, cardiogenic shock, cigarette smoking, EF, hemoglobin, heating season, multivessel CAD, prior MI, SBP, WBC										
12-month MI	0.85 (0.64–1.14)	0.27	0.99 (0.72–1.37)	0.96	0.79 (0.59–1.04)	0.1	0.76 (0.56–1.02)	0.07	0.99 (0.73–1.33)	0.94
Covariates used in the model: year of the admission, creatinine, cardiogenic shock, cigarette smoking, diabetes mellitus, heating season, EF, hemoglobin, multivessel CAD, prior MI, WBC										
12-month stroke	1.28 (0.67–2.44)	0.45	1.19 (0.57–2.46)	0.64	1.58 (0.86–2.88	0.14	1.13 (0.59–2.14)	0.10	0.68 (0.30–1.57)	0.37
Covariates used in the model: year of the admission, age, female, hemoglobin, heating season, EF, multivessel CAD, prior MI										
12-month ACS-driven PCI	1.03 (0.77–1.37)	0.84	1.01 (0.73–1.40)	0.95	0.87 (0.66–1.16)	0.35	0.91 (0.68–1.22)	0.53	1.17 (0.88–1.57)	0.28
Covariates used in the model: year of the admission, cardiogenic shock, diabetes mellitus, female, hemoglobin, heating season, multivessel CAD, prior MI, WBC.										

## Data Availability

The data on air pollution are available on: https://powietrze.gios.gov.pl/pjp/archives.

## Supplementary Materials

The following are available online at https://www.mdpi.com/article/10.3390/jcm11030484/s1, Table S1: The influence of elevated concentration of air pollutants on in-hospital outcomes in study groups.
