# Supplementary material for: The Impact of Short-Term Outdoor Air Pollution on Clinical Status and Prognosis of Hospitalized Patients with Coronary Artery Disease Treated with Percutaneous Coronary Intervention
Source: J Clin Med. 2022 Jan 18;11(3):484. doi: 10.3390/jcm11030484 (PMC8836379; doi:10.3390/jcm11030484)
Supplement: Supplementary file 1 [file jcm-11-00484-s001.zip › jcm-1370114-supplementary.pdf]

Table S1. The influence of elevated concentration of air pollutants on in-hospital outcomes in study groups.

		PM10			SO_2			NO			NO_2			O_3		
		OR	95% CI	*p*	OR	95% CI	*p*	OR	95% CI	*p*	OR	95% CI	*p*	OR	95% CI	*p*
Death	CCS	1.32	0.58–3.01	0.51	0.87	0.34–2.17	0.77	1.03	0.45–2.36	0.95	0.68	0.26–1.80	0.44	0.47	0.16–1.34	0.16
	ACS	0.97	0.71–1.32	0.84	1.05	0.71–1.55	0.81	0.75	0.50–1.14	0.18	0.88	0.59–1.31	0.52	0.83	0.55–1.26	0.39
MI	CCS	0.97	0.39–2.42	0.95	0.94	0.34–2.57	0.90	0.31	0.07–1.31	0.11	0.70	0.23–2.07	0.51	0.44	0.13–1.50	0.19
	ACS	1.15	0.65–2.03	0.63	1.03	0.58–1.83	0.92	0.93	0.50–171	0.80	0.82	0.44–153	0.54	1.24	0.69–2.21	0.47
Cardiac arrest	CCS	0.98	0.40–2.41	0.96	0.94	0.40–2.21	0.90	0.77	0.33–1.76	0.53	0.65	0.27–1.56	0.33	0.47	0.18–1.20	0.11
	ACS	1.01	0.85–1.20	0.90	1.06	0.81–1.48	0.55	0.84	0.62–1.16	0.29	1.08	0.81–1.46	0.59	0.96	0.71–1.29	0.77

Abbreviations: ACS, acute coronary syndrome; CCS, chronic coronary syndrome; MI, myocardial infarction; NO, nitrogen monoxide; NO_2, nitrogen dioxide; O_3, daily maximum of 8-hour mean of ozone; PM10, particulate matter with aerodynamic diameter <10 μm; SO_2, sulphur dioxide.
